# Different local anesthetic volumes in pericapsular nerve group block for hip fracture patients

**DOI:** 10.55730/1300-0144.6038

**Published:** 2025-04-22

**Authors:** Yusuf ÖZGÜNER, Savaş ALTINSOY, Gökçen KÜLTÜROĞLU, Sevda Gökçe GÜRPINAR, Derya ÖZKAN, Jülide ERGİL, Erbil AYDIN

**Affiliations:** 1 Department of Anesthesiology and Reanimation, Ankara Etlik City Hospital, Ankara, Turkiye; 2 Department of Orthopedics and Traumatology, Ankara Training and Research Hospital, Ankara, Turkiye

**Keywords:** Hip fractures, pain management, pericapsular nerve group block, regional anesthesia

## Abstract

**Background/aim:**

The pericapsular nerve group (PENG) block is an effective method for pain management in patients with hip fractures. In our study, we compared three different local anesthetic volumes of the PENG block in patients who underwent surgery for hip fracture.

**Materials and methods:**

Patients who underwent surgery with spinal anesthesia for intertrochanteric femur fractures (60 patients) were divided into three groups based on the volume of local anesthetic administered: Group 1 (20 mL), Group 2 (30 mL), and Group 3 (40 mL). Postoperative patient-controlled analgesia was initiated. Postoperative tramadol consumption, rest and movement pain scores, and the duration of motor block were monitored.

**Results:**

We found that Group 2 (118 ± 35.48 mg) and Group 3 (115 ± 42.98 mg) had reduced tramadol consumption compared to Group 1 (151 ± 31.43 mg) (p < 0.05). However, Group 3 (161 ± 18.6 min) had a longer duration of motor block (time to reach a Bromage score of 0) compared to Group 1 (132.25 ± 13.71 min) and Group 2 (143.5 ± 19.54 min) (p < 0.05).

**Conclusion:**

We found that the 30 mL and 40 mL volumes in the PENG block resulted in lower tramadol consumption compared to the 20 mL volume. We believe that the 30 mL volume is the most appropriate option among the three volumes, as it provides similar analgesic efficacy to the 40 mL volume but causes less motor block.

## 1. Introduction

Hip fractures are frequently observed in the elderly patient population [1]. Patients with hip fractures experience severe pain that intensifies with movement, resulting in decreased mobility [2]. This reduced mobility can lead to cardiopulmonary complications and prolonged hospitalization [3].

Various regional anesthesia methods, such as the erector spinae plane, femoral nerve, fascia iliaca, pericapsular nerve group (PENG) block, and psoas compartment blocks, have been employed for postoperative pain management in patients with hip fractures [4]. Girón-Arango and colleagues were the first to describe the PENG block, which is effective in blocking the obturator and femoral nerves [5]. The PENG block was initially described using 20 mL of local anesthetic (LA). Subsequent studies have explored the use of higher volumes, with some employing volumes as high as 40 mL [5–8]. This study investigated the clinical effects of administering 20 mL, 30 mL, and 40 mL of local anesthetic for the PENG block in patients with hip fractures.

The primary aim of our study was to investigate the effect of different volumes of LA on postoperative opioid consumption. As a secondary objective, we aimed to evaluate patients’ pain levels and the duration of motor block.

## 2. Materials and methods

### 2.1. Ethical statement

This single-center randomized controlled study complied with the ethical standards of the Helsinki Declaration-2013. Ethics committee approval (115/07) was obtained. Written informed consent was obtained from all the participants.

### 2.2. Study design

Patients over 18 years of age who consented to participate and had surgery for intertrochanteric femur fractures with an American Society of Anesthesiologists (ASA) classification of I, II, or III were included. Patients with coagulopathy, peripheral neuropathy, LA allergy, chronic opioid use, or intraoperative complications were excluded from the study.

**Figure 1:**
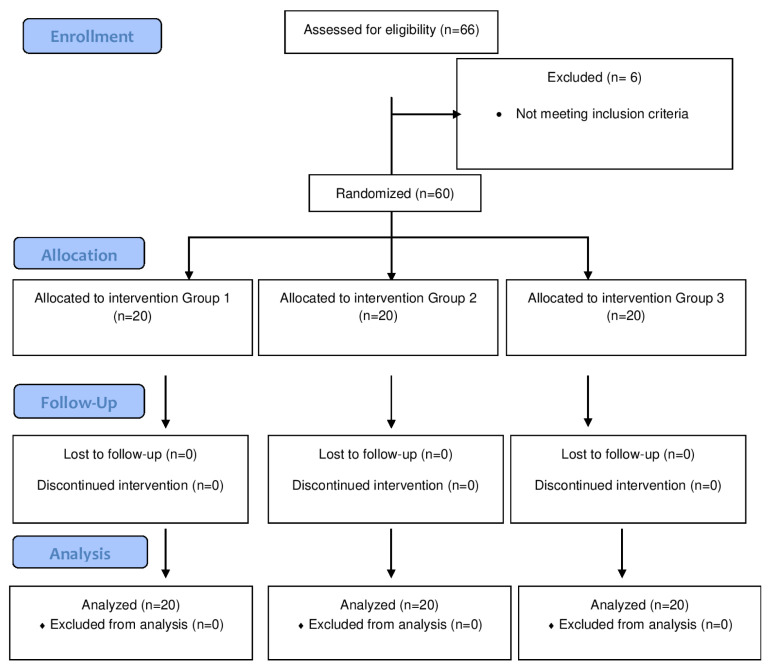
CONSORT diagram of the study.

A power analysis was performed based on the results of a preliminary study. Power analysis data are available in the statistical analysis section. Sixty-six patients were included in the study. Six patients in whom spinal anesthesia was unsuccessful were excluded from the study. Consequently, 60 patients were analyzed. Patients were randomly assigned to one of three groups (n = 20 each) using sealed envelopes: Group 1 (20 mL), Group 2 (30 mL), and Group 3 (40 mL) (Figure 1).

In the literature, the PENG block was initially performed using a 20 mL volume [5]. Subsequent studies have reported the use of higher volumes, such as 30 mL and 40 mL [6–7]. Accordingly, the patient groups in this study were assigned to receive 20 mL, 30 mL, or 40 mL of local anesthetic.

A blinded anesthetist monitored the patients postoperatively. The primary outcome measure of the study was total tramadol consumption within the first 24 h postoperatively, while the secondary outcome measures were postoperative NRS and Bromage scores.

### 2.3. Anesthesia and block procedures

For patient monitoring, all groups utilized electrocardiography (ECG), pulse oximetry (SpO_2_), noninvasive blood pressure, temperature, and urine output monitoring. All patients received spinal anesthesia with 10 mg of hyperbaric bupivacaine in the lateral position, 30 min after PENG block administration. Surgery commenced after confirmation of spinal anesthesia. Balanced fluid resuscitation was maintained intraoperatively for all patients.

**Figure 2:**
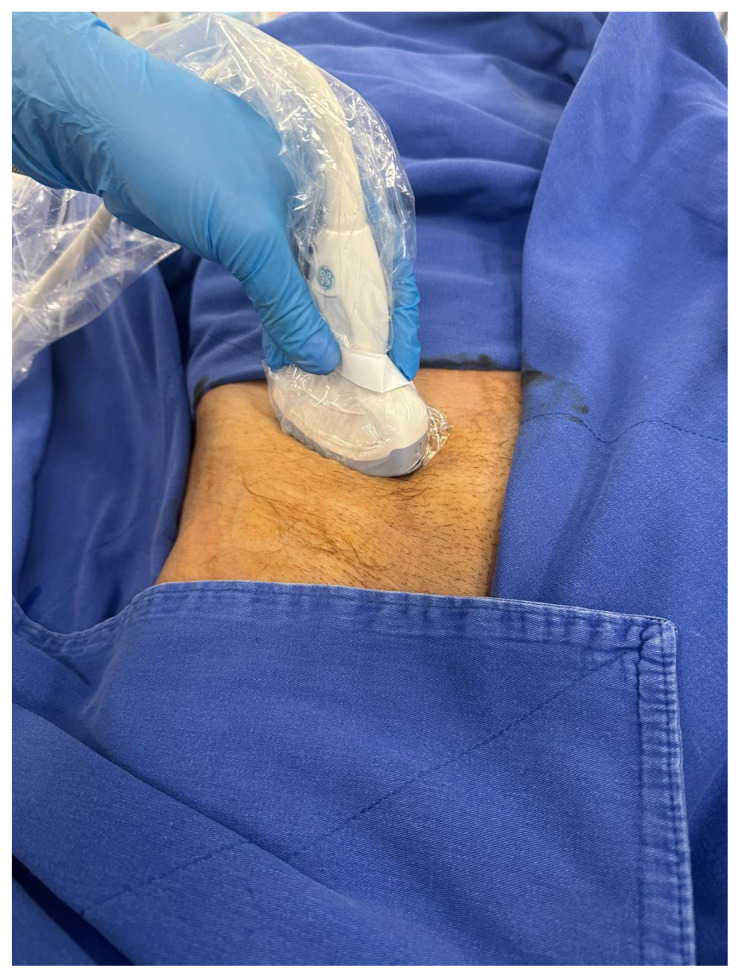
Ultrasonography probe position.

**Figure 3:**
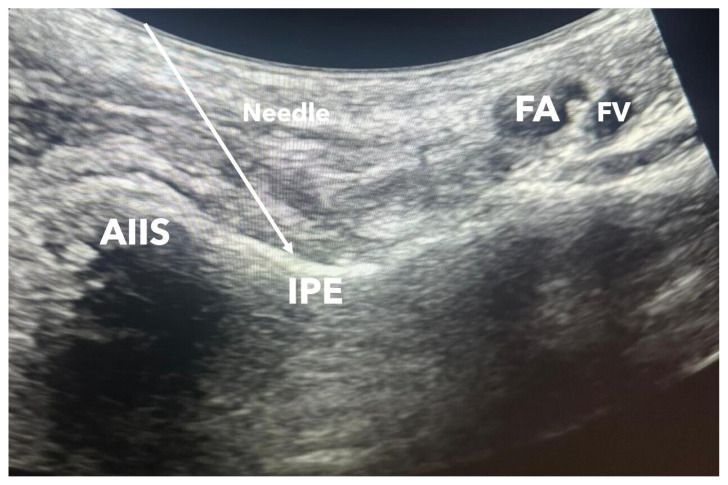
Sonoanatomy of PENG block. AIIS: anterior inferior iliac spine, FA: femoral artery, FV: femoral vein, IPE: iliopubic eminence.

After monitoring, with the patient in the supine position, a convex ultrasound probe (2–5 MHz) was placed on the anterior superior iliac spine (ASIS) and directed medially until the femoral artery was visualized (Figure 2). The iliopubic eminence (IPE), iliopsoas muscle and tendon, femoral artery, and the iliacus muscle were identified. A 22-gauge, 80 mm block needle was inserted between the psoas tendon and the IPE, and LA was administered with intermittent aspiration. Local anesthetic administration included 20 mL in Group 1, 30 mL in Group 2, and 40 mL in Group 3 (0.25% bupivacaine) (Figure 3). Sensory and motor assessments were conducted 15 and 30 min after block administration. Motor function was evaluated using the straight leg raise test.

### 2.4. Postoperative pain management

Postoperatively, patient-controlled analgesia with intravenous tramadol was initiated for all patients (bolus dose: 20 mg; lockout time: 20 min). Postoperative tramadol consumption and pain scores were recorded by a blinded anesthetist. Rescue analgesia was administered to patients with an NRS score of 4 or higher (dexketoprofen 50 mg IV). Postblock assessments were carried out at 15 and 30 min. patient-controlled analgesia (PCA) consumption, static and dynamic numerical rating scale (NRS) pain scores, and Bromage scores were recorded at 0, 2, 4, 8, 12, and 24 h postoperatively. Dynamic pain scores were recorded using the straight leg raise test.

### 2.5. Statistical analysis

A preliminary study was conducted, and the sample size was calculated based on our primary aim, which was the 24 h mean tramadol consumption. The analysis was performed using G*Power software. A total of 15 patients were included in the preliminary study, with five patients from each group. The 24 h mean tramadol consumption (mean ± SD) was as follows: Group 1 (152 ± 48.16 mg), Group 2 (120 ± 24.49 mg), and Group 3 (112 ± 41.47 mg). A one-way ANOVA test (fixed effects, omnibus) was used with an α-error of 0.05, 80% power, and an effect size of 0.42 for the three groups. According to the preliminary study, the total sample size required was calculated as 57 patients. Taking potential dropouts into account, 66 patients were included in the study.

Statistical analysis was performed using SPSS software (version 21.0; IBM Corp., Armonk, NY, USA). The chi-square test (for categorical variables), one-way ANOVA (for continuous variables with normal distribution), and Kruskal–Wallis test (for continuous variables with nonnormal distribution) were employed in this study. A p-value of <0.05 was considered statistically significant. The Tukey test was used for multiple comparisons between the groups.

## 3. Results

None of the 60 patients included in the study was excluded during the postoperative period. The study was completed with 20 patients in each group (Figure 1).

The demographic and clinical characteristics of the groups were similar. Preoperative and postoperative hemoglobin values, intraoperative blood loss, and fluid volumes administered were also comparable across all groups (Table 1). The duration of surgery was 91 ± 10.86 min for Group 1, 91.95 ± 9.57 min for Group 2, and 90.7 ± 11.96 min for Group 3. The surgery durations were similar between the groups (p = 0.930) (Table 1). During spinal anesthesia, positional pain occurred in four patients (Group 1: 3; Group 2: 1) (p = 0.153). Significant differences in NRS scores were observed at B15 (dynamic), at the 4th hour during movement, and at the 8th and 12th hours (Tables 2–3). Differences in PCA consumption were noted at the 4th, 8th, 12th, and 24th hours among the groups (Table 4). Rescue analgesic consumption was similar across the groups (Group 1: 5; Group 2: 4; Group 3: 4). Sensory examinations were conducted at 15 and 30 min after the block was administered. Sensory block levels ranged from T6–T12 at the highest to L1 at the lowest on the blocked side. The postoperative time to achieve a Bromage score of 0 was 132.25 ± 13.71 min for Group 1, 143.5 ± 19.54 min for Group 2, and 161 ± 18.6 min for Group 3 (Table 5). Knee extension weakness was observed in six patients (Group 2: 2; Group 3: 4) at the 2nd and 4th hours (p = 0.108). No knee extension weakness was observed in any patient at the 8th, 12th, and 24th hours (Table 5).

## 4. Discussion

In this study, we investigated the effects of the PENG block using different volumes of 0.25% bupivacaine. We found that Group 2 and Group 3 had lower pain scores and reduced opioid consumption compared to Group 1. However, Group 3 had a longer duration of motor block (time to reach a Bromage score of 0) compared to the other groups. Additionally, we observed differences between Group 2 and Group 3 in terms of dynamic pain scores at 15 min and opioid consumption at the 4th hour postoperatively.

Various studies in the literature have investigated the efficacy of different volumes of LA in numerous peripheral nerve blocks. The literature indicates that increased LA volumes in erector spinae plane blocks and fascia iliaca compartment blocks provide better analgesic efficacy [9,10]. Zhang and colleagues reported that higher volumes in supraclavicular brachial plexus blocks led to a faster onset time without affecting the duration of analgesia. However, higher volumes were associated with a significantly increased incidence of diaphragmatic paralysis [11]. In another study, Serradel and colleagues reported that different volumes provided similar block efficacy across all groups in axillary plexus block [12]. However, there is currently no definitive recommendation regarding the optimal volume for the PENG block. In this study, Group 1 exhibited higher pain scores and greater opioid consumption compared to the other groups. Additionally, patients in the 40 mL volume group demonstrated higher dermatomal sensory block levels and lower pain scores at 15 min postblock compared to the other groups. Despite lower NRS scores and opioid consumption at 15 min and at the 4th hour postblock in Group 3 compared to Group 2, both groups had similar pain scores and opioid consumption in the subsequent hours. We found that the higher volume of LA used in Group 3 led to a faster onset of the block, but did not result in differences in analgesic efficacy during the later periods. Given that the 30 mL and 40 mL volumes of the PENG block provide similar postoperative analgesic efficacy, we think that a 30 mL volume may be sufficient. In the studies conducted by Zhang and Serradell, the differing results may be attributed to the more limited anatomical spread of LA in the brachial plexus region, which inherently involves a smaller fascial compartment. In contrast, the PENG block targets a broader fascial plane, which may explain why the volume of LA plays a more significant role in its effectiveness in our study. Therefore, determining the minimum effective volume is of importance in this context. Unlike previous studies, although higher volumes of LA were associated with improved analgesic efficacy, we observed a notable increase in undesirable effects, such as motor weakness, which warrants careful consideration.

In studies where the PENG block was performed using local anesthetics at concentrations of 0.5% and 0.75%, motor block has been reported in patients [13–15]. It is believed that motor block may occur in patients undergoing PENG block due to the spread of LA around the femoral nerve, obturator nerve, and sciatic nerve [16–18]. In our study, Group 3 had a longer time to reach a Bromage score of 0 compared to the other groups. However, we found that this duration was similar in Groups 1 and 2. Ciftci et al. reported in a cadaveric study that the 30 mL volume showed a greater spread pattern compared to the 20 mL volume [19]. Leurcharusmee et al. investigated the volume that preserves the femoral nerve in a cadaveric study and reported that the maximum volume was 13.2 mL [20]. We think that the concentration and volume of LA used influence the occurrence of motor block in the PENG block. We think that the higher incidence of motor block in patients who received 40 mL of LA in our study is due to the greater spread of LA around the femoral nerve associated with larger volumes. Motor block has been reported even in studies where the LA volume was 20 mL. Therefore, we think that reducing the LA concentration during PENG block application may reduce the likelihood of motor block.

Prolonged immobilization in patients with hip fractures has been reported to increase the incidence of cardiopulmonary complications, such as embolism and pneumonia, as well as infections [21,22]. Moreover, higher mortality rates have been observed in patients who are unable to walk and do not receive early rehabilitation [23]. Inadequately managed pain can also lead to immobilization. Therefore, we emphasize the importance of achieving optimal analgesic efficacy with minimal motor weakness. We think that utilizing the volume of LA that provides the best analgesic effect with the least motor blockade in the PENG block may help reduce potential postoperative complications and facilitate early rehabilitation. In our study, the use of 30 mL of LA resulted in a motor block duration comparable to 20 mL, with similar analgesic efficacy when compared to the 40 mL volume. In light of these findings, we consider 30 mL to be the optimal volume for the PENG block among the three groups.

Our study has several limitations. First, due to the use of spinal anesthesia, we could not assess the sensory and motor block effects of the PENG block alone in the early postoperative period. Second, we were unable to evaluate long-term clinical outcomes due to the follow-up period being limited to 24 h postoperatively.

In conclusion, the PENG block is an effective method for pain management in patients with hip fractures. In this study, we found that the PENG block performed with 30 mL and 40 mL of LA was superior in pain management compared to the 20 mL volume. However, we found that there was a higher incidence of motor block with the 40 mL volume. Therefore, we think that 30 mL of 0.25% bupivacaine is more appropriate for the PENG block due to its similar analgesic efficacy and lower incidence of motor block. Additionally, we think that further studies investigating different volumes and concentrations of LA are needed to optimize the PENG block.

## Figures and Tables

**Table 1:** Demographic and clinic characteristics.

	Group 1 n = 20	Group 2 n = 20	Group 3 n = 20	p
Age (year)	74.25 ± 7.33	75.25 ± 8.05	74.6 ± 7.77	0.918
Sex (n): female/male	13/7	14/6	13/7	0.928
BMI (kg/m^2^)	24.26 ± 2.75	25.31 ± 3.82	25.09 ± 2.88	0.552
ASA score (I/II/III)(n)	1/7/12	1/4/15	1/6/13	0.882
FRAIL scale score (0/1/2/3) (n)	2/1/9/8	2/1/7/10	1/2/9/8	0.958
Duration of surgery (minutes)	91 ± 10.86	91.95 ± 9.57	90.70 ± 11.96	0.930
Preoperative hemoglobin (g/dL)	11.84 ± 1.43	11.73 ± 1.36	11.89 ± 1.26	0.930
Postoperative hemoglobin (g/dL)	11.08 ± 1.32	10.43 ± 1.14	10.96 ± 1.21	0.211
Intraoperative blood loss (mL)	112 ± 65.74	92.5 ± 74.82	125 ± 67.86	0.338
Volume of fluid administered (mL)	1549 ± 315.59	1502.25 ± 178.21	1627.5 ± 335.79	0.380

Values are presented as mean ± SD and numbers. Group 1: 20 mL local anesthetic, Group 2: 30 mL local anesthetic, Group 3: 40 mL local anesthetic. n: number, BMI: Body mass index, ASA: American Society of Anesthesiologists.

**Table 2:** Static pain ccores.

	Group 1	Group 2	Group 3	p
Preoperative NRS	3.5 (5)	3.5 (4)	4 (4)	0.885
Postblock 15th minute	2 (4)	1.5 (3)	1 (3)	0.111
Postblock 30th minute	1 (2)	1 (2)	1 (2)	0.285
NRS 0th hour	1 (3)	0 (2)	0 (2)	0.471
NRS 2nd hour	1 (2)	0 (2)	0 (1)	0.204
NRS 4th hour	1.5 (4)	1 (4)	1 (4)	0.398
NRS 8th hour	2 (2)	1 (3)	1 (3)	**<0.001** *
NRS 12th hour	2 (2)	1.5 (4)	1 (2)	**<0.001** †
NRS 24th hour	1 (3)	1 (3)	1 (3)	0.830

Values are given as median (range). Group 1: 20 mL local anesthetic, Group 2: 30 mL local anesthetic, Group 3: 40 mL local anesthetic. NRS: Numeric rating score. Values shown in bold indicate statistically significant differences.

* There was a difference between Group 1 and Group 3.

† There was a difference in Group 1 compared to Groups 2 and 3.

**Table 3:** Dynamic pain scores.

	Group 1	Group 2	Group 3	p
Preoperative NRS	8.5 (5)	8 (4)	8.5 (3)	0.908
Postblock 15th minute	3 (4)	3 (4)	1.5 (4)	**<0.001** *
Postblock 30th minute	2 (2)	2 (3)	1 (2)	0.121
NRS 0th hour	2 (3)	2 (3)	1 (3)	0.688
NRS 2nd hour	2 (3)	2.5 (4)	2 (3)	0.343
NRS 4th hour	3.5 (3)	3 (5)	2 (4)	**<0.05** †
NRS 8th hour	3 (3)	2 (4)	2 (3)	**<0.001** ‡
NRS 12th hour	4 (2)	3 (4)	2.5 (3)	**<0.001** ‡
NRS 24th hour	3 (4)	3 (5)	3 (5)	0.845

Values are given as median (range). Group 1: 20 mL local anesthetic, Group 2: 30 mL local anesthetic, Group 3: 40 mL local anesthetic. NRS: Numeric rating score. Values shown in bold indicate statistically significant differences.

* There was a difference in Group 3 compared to Groups 1 and 2.

† There was a difference between Group 1 and Group 3.

‡ There was a difference in Group 1 compared to Groups 2 and 3.

**Table 4:** Comparison of tramadol consumption of groups.

	Group 1	Group 2	Group 3	p
0th hour	7 ± 13.41	5 ± 11.01	6 ± 9.40	0.858
2nd hour	27 ± 22.73	20 ± 24.27	20 ± 20.51	0.530
4th hour	61 ± 23.81	52 ± 26.27	31 ± 21.98	**<0.05** *
8th hour	91 ± 27.89	68 ± 27.83	65 ± 41.88	**<0.05** †
12th hour	128 ± 28.58	92 ± 35.18	91 ± 41.78	**<0.05** †
24th hour	151 ± 31.43	118 ± 35.48	115 ± 42.98	**<0.05** †

Tramadol consumption values according to postoperative hours are given as mean ± SD (mg). Group 1: 20 mL local anesthetic, Group 2: 30 mL local anesthetic, Group 3: 40 mL local anesthetic. Values shown in bold indicate statistically significant differences

* **:** There was a difference in Group 3 compared to Groups 1 and 2.

† **:** There was a difference in Group 1 compared to Groups 2 and 3.

**Table 5:** Dermatome and motor blockade times between groups.

	Group 1 (20)	Group 2 (20)	Group 3 (20)	p
Sensory blockade at T12 level at 15 min after blockade (n)	6	10	15	**p<0.05** *
Sensory blockade at T12 level at 30 min after blockade (n)	14	17	19	p=0.102
Knee extension weakness(n)	0	2	4	p=0.108
Time to Bromage score 0 (minute)*	132.25 ± 13.71	143.5 ± 19.54	161 ± 18.6	**p<0.05** *

Values are given as number and mean ± SD. Group 1: 20 mL local anesthetic, Group 2: 30 mL local anesthetic, Group 3: 40 mL local anesthetic. n: number. Values shown in bold indicate statistically significant differences.

* **:** There was a difference in Group 3 compared to Groups 1 and 2.
